# Chemodiversity of Brevetoxins and Other Potentially Toxic Metabolites Produced by *Karenia* spp. and Their Metabolic Products in Marine Organisms

**DOI:** 10.3390/md19120656

**Published:** 2021-11-24

**Authors:** Vincent Hort, Eric Abadie, Nathalie Arnich, Marie-Yasmine Dechraoui Bottein, Zouher Amzil

**Affiliations:** 1 Laboratory for Food Safety, Pesticides and Marine Biotoxins Unit, ANSES (French Agency for Food, Environmental and Occupational Health and Safety), 94701 Maisons-Alfort, France; 2 MARBEC (MARine Biodiversity, Exploitation and Conservation), Université de Montpellier, CNRS, Ifremer, IRD, 34200 Sète, France; eric.abadie@ifremer.fr; 3 Risk Assessment Directorate, ANSES (French Agency for Food, Environmental and Occupational Health and Safety), 94701 Maisons-Alfort, France; nathalie.arnich@anses.fr; 4 Université Côte d’Azur, CNRS, UMR 7035 ECOSEAS, 06103 Nice, France; marie-yasmine.bottein@univ-cotedazur.fr; 5 Federative Research Institute—Marine Ressources, Université Côte d’Azur, CNRS, 06108 Nice, France; 6 Ifremer (French Research Institute for Exploitation of the Sea), 44311 Nantes, France

**Keywords:** *Karenia* spp., marine biotoxins, brevetoxins, metabolic products, shellfish, marine organisms

## Abstract

In recent decades, more than 130 potentially toxic metabolites originating from dinoflagellate species belonging to the genus *Karenia* or metabolized by marine organisms have been described. These metabolites include the well-known and large group of brevetoxins (BTXs), responsible for foodborne neurotoxic shellfish poisoning (NSP) and airborne respiratory symptoms in humans. *Karenia* spp. also produce brevenal, brevisamide and metabolites belonging to the hemi-brevetoxin, brevisin, tamulamide, gymnocin, gymnodimine, brevisulcenal and brevisulcatic acid groups. In this review, we summarize the available knowledge in the literature since 1977 on these various identified metabolites, whether they are produced directly by the producer organisms or biotransformed in marine organisms. Their structures and physicochemical properties are presented and discussed. Among future avenues of research, we highlight the need for more toxin occurrence data with analytical techniques, which can specifically determine the analogs present in samples. New metabolites have yet to be fully described, especially the groups of metabolites discovered in the last two decades (e.g tamulamides). Lastly, this work clarifies the different nomenclatures used in the literature and should help to harmonize practices in the future.

## 1. Introduction

The genus *Karenia* belongs to the clade of the alveolates (with the ciliates and the apicomplexa). It includes 12 known marine species from oceanic and coastal areas. These eukaryotes were formerly classified as microalgae, and belong to the group of photosynthetic protists (autotrophs) [1]. Among the genus *Karenia*, some species of this dinoflagellate can proliferate and form dense blooms that cause multiple dysfunctions of the hydrosystem, but also have a strong toxic potential for organisms in the food web [2,3]. These massive blooms, referred as “red tides”, can be visible and color the water. Brevetoxins (BTXs), which are produced by *Karenia brevis* (C.C. Davis) Gert Hansen and Moestrup, 2000, belong to the main group of marine biotoxins associated with these events. These neurotoxins can cause significant mortality in fish, sea birds, and marine mammals [4,5,6]. Humans can also be exposed to BTX through food, leading to human poisoning, called neurotoxic shellfish poisoning (NSP), inhalation or contact (skin or mucous membranes) [7,8]. Shellfish consumption is the main cause of NSP. Symptoms of NSP include nausea, vomiting, diarrhea, paresthesia, cramps, bronchoconstriction, paralysis, seizures and coma. Inhalation or contact exposure can result in irritant effects. To date, NSP events have been limited to the Gulf of Mexico, the east coast of the United States of America (U.S.A.), and New Zealand [9]. No cases of NSP have been reported in Europe to date; however, a recent study has shown the presence of these toxins in shellfish from the French Mediterranean Sea, raising the question of the potential emergence of this group of toxins in areas that have been preserved to date [10,11]. *Karenia* spp. can also produce the following groups of potentially toxic metabolites in addition to the BTXs: hemi-brevetoxins, brevenals, brevisamides, brevisins, tamulamides, gymnocins, gymnodimines, brevisucenals, and brevisulcatic acids [12].

The use of systematic nomenclature to refer to these metabolites is cumbersome, considering their molecular weight and the large number of analogs. In recent decades, different nomenclatures have been used to name the toxins produced by *Karenia* spp., leading to a large number of names, sometimes for the same analog, especially for brevetoxins. One reason for this proliferation of names is that isolation and toxicology studies were sometimes carried out from the causative organisms, before pure materials were available and the structures of the metabolites were elucidated [13]. Moreover, one of the earliest nomenclatures was based on the name of the producer identified at that time: *Gymnodinium breve* C.C. Davis, 1948. For example, “GB-1 toxin” was the name attributed to BTX-1. In 1979, *Gymnodinium breve* was taxonomically reclassified to *Ptychodiscus brevis* (C.C. Davis) K.A.Steidinger, 1979. This change led to a nomenclature revision of brevetoxins, which became *Ptychodiscus brevis* toxins, with the abbreviation PbTx [14]. Eventually, in 2000, the brevetoxin producer was named *Karenia brevis* (*K. brevis*) by Hansen and Moestrup [15]. The term brevetoxin is the only name that has withstood the test of time. BTX is now widespread and taken up not only for recently isolated metabolites, but also for the first discovered metabolites, which were initially named following another nomenclature [10,11,16,17,18,19]. Despite the harmonization of the term BTX, several analogs with different names are still encountered, for several other reasons. Moreover, we should recall that this abbreviation is also shared with the batrachotoxins, a small group of steroidal alkaloids. 

Considering all the different groups of metabolites produced by *Karenia* spp., accumulation and biotransformation by marine organisms has been investigated in depth for BTXs and gymnodimines (GYMs), contrary to the other groups [4,17,18,20,21,22,23,24,25,26,27,28,29,30,31,32,33,34,35,36,37,38,39,40,41,42,43,44,45,46,47]. In 1992–1993, a major NSP outbreak occurred in New Zealand after a *K. brevis* bloom. A total of 180 people were intoxicated after shellfish consumption, with a toxin profile dominated by the analogue BTX-2. An analysis of the shellfish samples collected during this NSP event, combined with the implementation of specific analytical methods (particularly physicochemical techniques), led to the first evidence suggesting BTX metabolism in shellfish [20,21,31,33,36,37,38,40]. BTX-2, BTX-3 and BTX-B5, known to be produced by *K. brevis*, were detected in cockles, mussels and oysters collected during this event (*Austrovenus stutchburyi*, *Perna canaliculus* and *Crassostrea gigas*, respectively) [20,21,31,33]. Metabolites that were not identified from *K. brevis* natural blooms or cultures at that time were also isolated from these shellfish, particularly amino acid conjugates (taurine-BTX-B and cysteine-BTX-B sulfoxide, also called BTX-B1 and BTX-B2, respectively) and fatty acid conjugates (oxidized open D-ring tetradecanoyl-BTX-2 and oxidized open D-ring hexadecanoyl-BTX-2), and sometimes a combination of these transformations (N-tetradecanoyl-cysteine-BTX-B sulfoxide, also called BTX-B4). These preliminary results suggested the metabolism processes of BTXs in shellfish, and further investigations followed, with different shellfish species and other marine organisms either naturally exposed to *K. brevis*, in cultures, or to pure toxins [17,22,23,30,34,35,39,48,49,50]. BTX profiles varied depending on the species due to different rates of accumulation and elimination. Consequently, their toxicity fluctuates over time, based on the toxin profile. These studies also allowed for the identification of numerous new metabolites. For many of them, chemical structure elucidation relied on a stringent study of their mass spectrometry fragmentation profiles. Confirmation of these structures with more thorough elucidation tools, such as nuclear magnetic resonance (NMR), would require large quantities of samples for isolation and purification purposes, making this goal difficult to achieve.

The aim of this review is to summarize the knowledge collected in recent decades about the potentially toxic metabolites produced by *Karenia* spp. or reported in other marine organisms. Therefore, metabolites obtained by chemical synthesis (partially or fully from existing compounds) or from *in vitro* metabolism studies will not be considered [51,52,53,54,55]. We will present and discuss the structures and physicochemical properties of the metabolites reported in the literature. The accumulation and biotransformation of these potentially toxic metabolites by marine organisms will be discussed in depth and will help to identify and highlight future avenues of research. As previously mentioned, the use of different nomenclatures can be disruptive and it is not always easy to switch between the different names used, especially for BTXs. We will tackle this aspect by reporting all the names used in the literature and by preferentially using those which seem to achieve a better overall consistency.

## 2. Metabolites Produced by *Karenia* spp.

Among the different *Karenia* species reported, *K. brevis* is known to produce BTXs, brevisin, brevisamide, brevenals and tamulamides, whereas brevisulcenals and brevisulcatic acids can be produced by *Karenia brevisulcata* (F.H. Chang, 1999) G. Hansen & Moestrup, 2000. Gymnocins and gymnodimine-A were discovered from cultures of *Karenia mikimotoi* (Miyake & Kominami ex Oda) G. Hansen & Moestrup, 2000 [24,56,57,58], whereas gymnodimine-B and -C were first isolated from *Karenia selliformis* A.J. Haywood, K.A. Steidinger & L. MacKenzie, 2004 [59]. All these groups of metabolites possess different structure skeletons that give them their physicochemical properties (Table 1). To allow for a better follow-up of the numerous compounds reported in this review, an identification number (ID) was attributed to each of them in the following parts of this article (text, tables and figures).

**Figure 1:**
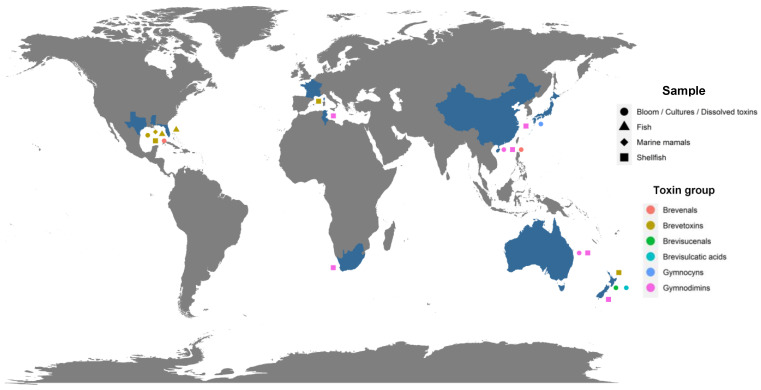
Geographical distribution of potentially toxic metabolites individually and formally identified (blue color for country), displayed by type of samples collected (forms) and by group to which they belong (colors of forms). Map generated using R software and the ggplot2 package [83,84].

The implementation of specific methods for the analysis of metabolites produced by *Karenia* spp. allowed for the identification of individual analogs belonging to several of these groups of toxins in Australia, Japan, New Zealand, the Gulf of Mexico and the South China Sea (Figure 1) [16,35,45,46,47,56,64,75,76,77,81,82].

### 2.1. Brevetoxins

**Figure 2:**
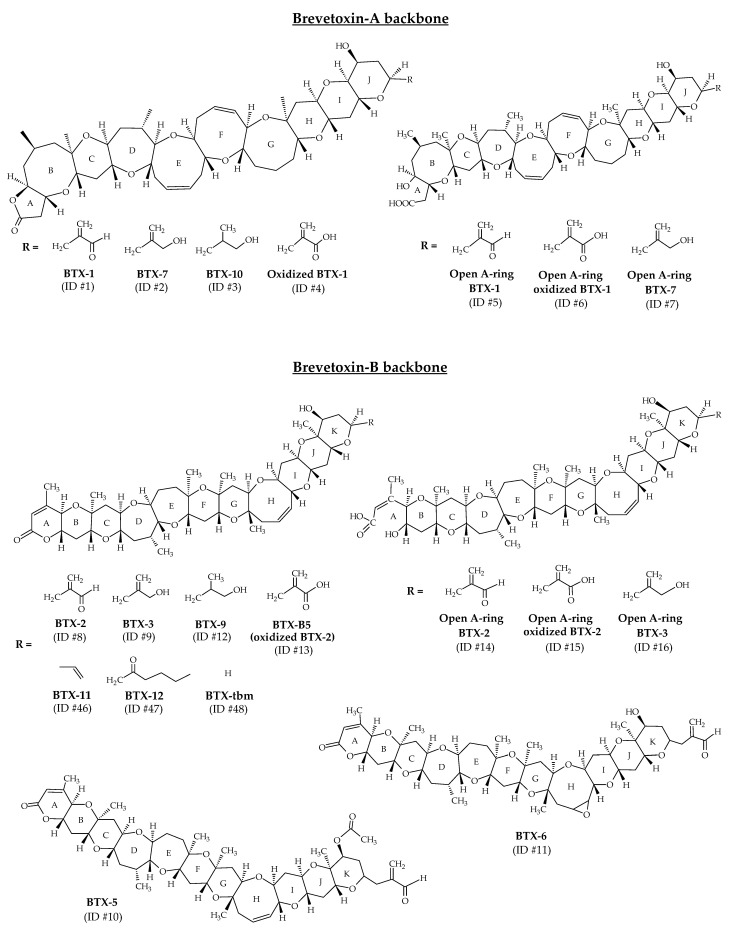
Chemical structures of brevetoxins (BTXs) identified from environmental samples and cultures of *Karenia brevis*.

BTXs are lipid-soluble and thermostable polycyclic polyethers produced by the dinoflagellate *Karenia brevis* [13]. In laboratory conditions, the production of BTX-2 (ID #8) has also been shown for *Karenia papilionacea* A.J. Haywood and K.A. Steidinger, 2004 [85]. These toxins are polyethers with ladder-like structures similar to ciguatoxins (CTXs) (produced by the dinoflagellate *Gamberdiscus*). All polyether compounds produced by *K. brevis* are thought to derive from polyepoxide precursors [86,87]. A wide range of BTXs have been identified from *K. brevis* cultures (Table 1). NMR and X-ray crystallography were the analytical techniques implemented to establish their structures. A-type and B-type BTXs (BTX-A and BTX-B) are the two base skeletons identified (Figure 2). BTX-1 (ID #1) and BTX-2 are the parent algal toxins of the BTX-A and BTX-B types, respectively [88]. The molecular formula of BTX-1 is C_49_H_70_O_13_ and its structure is composed of 10 rings [60,66]. The molecular formula of BTX-2 is C_50_H_70_O_14_ and its structure is composed of 11 rings [14,64]. Both structures carry a lactone function that is thought to be responsible for their biological activity [88]. BTX-2 is the major toxin isolated from *K. brevis* and the one produced by the largest number of species of the genus *Karenia* [88].

The structural elucidation of BTX-1 (ID #1) and BTX-2 (ID #8) facilitated the resolution of the structure of all subsequently discovered all related analogs (Figure 2). Each family consists of several compounds, differentiated by the “J-ring” in BTX-A (BTX-1, BTX-7, BTX-10 with respective ID of #1, #2, #3) and at the “K-ring” in BTX-B (BTX-2, BTX-3, BTX-5, BTX-6, BTX-9 with respective ID of #8, #9, #10, #11, #12) [13,14,35,60,61,65,66,67,89]. These different metabolites are reduced, acetylated or epoxidized forms of BTX-1 (ID #1) or BTX-2 (ID #2). Other analogs have been identified from *K. brevis* cultures and natural blooms, and correspond to the oxidized forms of BTX-1 (ID #4) and BTX-2 (BTX-B5, ID #13), and to the open forms of the first ring (A-ring) of BTX-1 (ID #5), BTX-2 (ID #14), BTX-3 (ID #16) and BTX-7 (ID #7) [35]. Open forms of oxidized BTX-1 (ID #6) and BTX-2 (ID #15) have also been reported (Figure 2). The structures of these open A-ring derivatives were postulated after a thorough study of their tandem mass spectrometry (MS/MS) fragmentations, but could not be elucidated with powerful techniques such as NMR. It is worth noting that some of these open A-ring derivatives were identified with greater abundance than their non-hydrolyzed counterparts.

In 2005, BTX-11 (ID #46), BTX-12 (ID #47) and BTX-tbm (BTX-2 decomposition product without the side chain tail; ID #48) were described as new toxins after their identification in cultures and in the field (Figure 2) [88,90]. Since then, these toxins were never detected. Moreover, several compounds initially identified as BTXs are now considered artifacts (not included in Table 1). BTX-8 (C_49_H_69_ClO_14_; also called BTX-C; ID #49) is generated during chloroform extraction of BTX-2 (ID #8) from *K. brevis* [35,88,91]. BTX-13 (ID #50) and BTX-14 (ID #51) are likely artifacts of acid-catalyzed methanol addition during purification protocols [88].

BTXs are neurotoxins that primarily target the voltage-gated Na+ channels (Na_V_) [11]. The neurological nature of the primarily symptoms observed in humans and animals can be explained by the specific affinity of BTXs for Na_V_ channel subtypes and the tissue distribution of Na_V_ channels. The symptoms also involve gastrointestinal and cardiovascular systems. Data on acute toxicity in animals are very limited. The only toxicity study by oral administration allows for an estimation of the median lethal doses (LD_50_) for BTX-2 and BTX-3 in female mice [14]. BTX-3 was about 10 times more toxic than BTX-2 by oral administration, with LD_50_ of 520 and 6600 µg/kg bw, respectively. However, by intraperitoneal injection, the two toxins were almost equipotent.

### 2.2. Hemibrevetoxins

**Figure 3:**
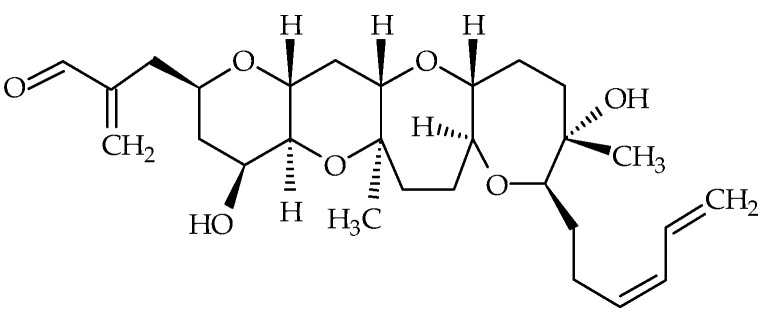
Chemical structure of hemibrevetoxin-B (ID #18).

Hemibrevetoxins (Hemi-BTX-A, -B, -C with respective ID of #17, #18, #19), cyclic ethers biosynthesized by *K. brevis*, were first isolated by Shimizu [68] in 1982 and were reported to have weak ichthyotoxicity. Later, Prasad and Shimizu elucidated the chemical structure of Hemi-BTX-B [69] (Figure 3). Its molecular formula is C_28_H_42_O_7_, and is about half that of BTXs, hence its name (Table 1). The authors suggested that hemibrevetoxins could play an important role in the biosynthesis of BTXs. They also noted cytotoxicity at a concentration of 5 µmol on cultured mouse neuroblastoma cells, and a characteristic rounding of cells, as for BTX-A and BTX-B. Hemi-BTX-A and –C were isolated; however, their chemical structures seem to have never been fully elucidated [68,69].

### 2.3. Brevenals

**Figure 4:**
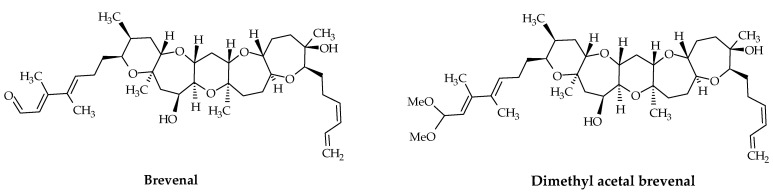
Chemical structures of brevenal (ID #20) and dimethyl acetal brevenal (ID #21).

In 2004, brevenal (ID #20) and its dimethyl acetal (ID #21) were first isolated and characterized from *K. brevis* cells collected in Florida, USA (Figure 4). These fat-soluble compounds, with molecular formulae C_39_H_60_O_8_ and C_41_H_66_O_9_, respectively, are composed of five ether rings and possess an aldehyde function [16,70,81]. Brevenals are not toxic for fish and antagonize the effects of BTXs. Both competitively displace BTX from its binding site in rat brain synaptosomes. Moreover, brevenal inhibits the bronchoconstriction induced by BTX-2 (ID #8) and BTX-3 (ID #9) in a sheep assay [90]. In 2006, the first synthesis of brevenal was accomplished [92]. This work led to a revision of the structure of brevenal, which corresponds to the C26 epimer of the initially proposed natural product structure (Figure 4).

### 2.4. Brevisamide

**Figure 5:**
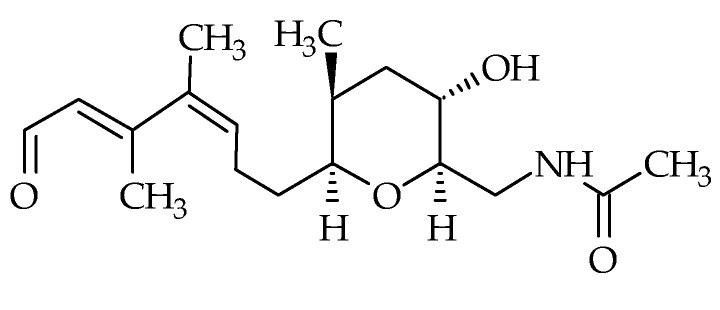
Chemical structure of brevisamide (ID #22).

In 2008, brevisamide (ID #22) was isolated from *K. brevis* [71]. Its molecular formula is C_18_H_29_NO_4_ and its structure is composed of a monocyclic ether possessing an amide function. It is also composed of a tetrahydropyran ring bearing a 3,4-dimethylhepta-2,4 dienal side chain (Figure 5). To date, no other analog has been discovered. Brevisamide was synthetized by several authors using different strategies [93,94,95]. Brevisamide may be the biosynthetic precursor of brevisin, based on their respective structures [95]. The toxicity of this metabolite remains unknown, due to the absence of any studies.

### 2.5. Brevisin

**Figure 6:**
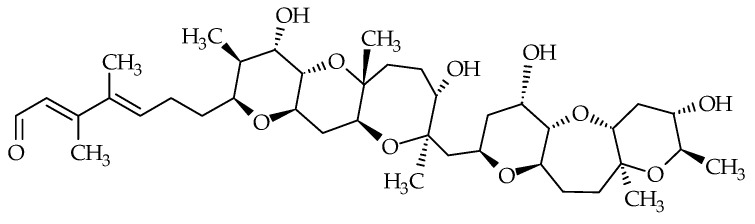
Chemical structure of brevisin (ID #23).

In 2009, brevisin (ID #23) was identified from *K. brevis* cells [72,96]. Its structure consists of two separate fused polyether ring assemblies linked by a methylene group. One of the polycyclic moieties was conjugated with an aldehyde side chain (Figure 6). Brevisin inhibits the binding of [^3^H]-BTX-3 on sodium channels in rat brain synaptosomes. In 2011, the total synthesis of brevisin was accomplished [97]. To date, studies regarding this secondary metabolite are limited and further investigation is required.

### 2.6. Tamulamides

**Figure 7:**
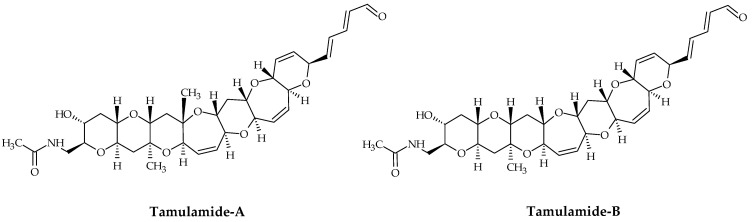
Chemical structures of tamulamide-A (ID #24) and tamulamide-B (ID #25).

Tamulamides, cyclic polyethers with a ladder-like chemical structure, were isolated from *K. brevis* cultures [73]. Both are composed of seven cyclic ethers with an amide and aldehyde side chain (Figure 7). The molecular formulae of Tamulamide-A (Tam-A; ID #24) and Tamulamide-B (Tam-B; ID #25) are C_35_H_45_NO_10_ and C_34_H_43_NO_10_, respectively (Table 1). Tam-A and Tam-B compete with [^3^H]-BTX-3 for its binding site on rat brain synaptosomes. Neither showed toxicity to fish at doses up to 200 nM and cause only slight bronchoconstriction in sheep pulmonary assays [73].

### 2.7. Gymnocins

**Figure 8:**
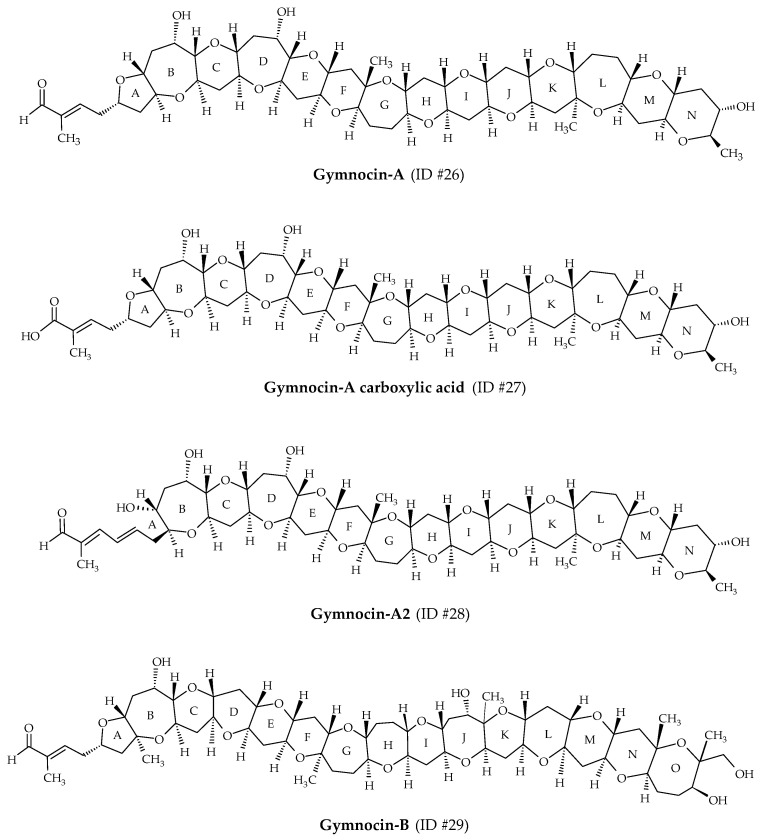
Chemical structures of gymnocins.

Gymnocins are polyethers biosynthesized by *K. mikimotoi*. Gymnocin-A (ID #26) was the first analog discovered from Japanese cultures of this dinoflagellate, isolated at Kushimoto Bay [56] (Figure 1). Structure elucidation revealed 14 saturated ether rings, bearing a 2-methyl-2-butenal side chain (Figure 8). In 2003, this analog was fully synthetized by Tsukano and Sasaki [98]. Gymnocin-A carboxylic acid (ID #27), Gymnocin-A2 (ID #28), Gymnocin-B (ID #29; composed of 15 adjacent rings ending with the same 2-methyl-2-butenal side chain as gymnocin-A) were isolated and elucidated from *K. mikimotoi* [57,58] (Table 1). Conventional fish assays showed that Gymnocin-A and Gymnocin-B are weakly toxic compared to BTX-B [56,57]. However, the authors highlighted that fish were directly exposed to dissolved gymnocins, whereas during red tide events, *K. mikimotoi* cells stuffed the fish gills, allowing direct contact of the gymnocins with the gills. The extremely low solubility of gymnocins to water could prevent them from reaching the fish gills during assays. This mechanism could be involved in the fish mortalities observed in the field, whereas conventional fish assays do not reveal significant toxicity. Gymnocin-A carboxylic acid and Gymnocin-A2 showed moderate cytotoxicity against P388 cells [58].

### 2.8. Gymnodimines (GYMs)

**Figure 9:**
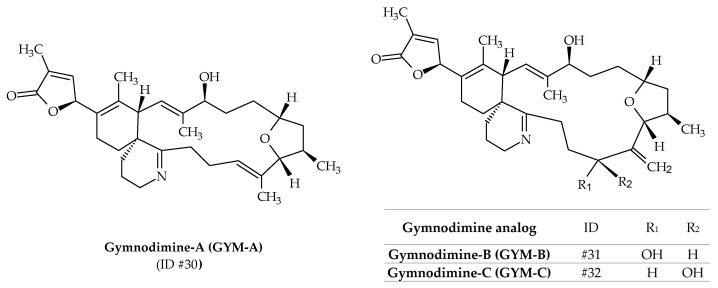
Chemical structures of gymnodimines (GYMs) identified from cultures of *Karenia selliformis*.

Initially, gymnodimine-A (GYM-A; ID #30) was isolated from extracts of New Zealand oysters (*Tiostrea chilensis*) [24] (Figure 1). Isolation of the active substance made it possible to determine its molecular formula as C_32_H_45_NO_4_ (Table 1), whereas its absolute stereochemistry was later determined by X-ray crystallography (Figure 9) [99]. The origin of this toxin was first attributed to the dinoflagellate *K. selliformis* for two reasons: efflorescence of the latter was observed at the same time; gymnodimine was isolated in cultures of this species [100]. Two other analogs are known to be produced by *K. selliformis*: GYM-B (ID #31) and GYM-C (ID #32); whereas several others (12-methyl-GYM-A, 12-methyl-GYM-B, GYM-D, 16-desmethyl-GYM-D, GYM-E and more than 30 related gymnodimine-like compounds) have only been identified from *Alexandrium ostenfeldii* or *Alexandrium peruvianum* [59,87,101,102,103,104]. The structure of GYM-B is similar to GYM-A, but contains an exocyclic methylene at position C17 and an allylic hydroxyl group at position C18 (Figure 9). GYM-C is an oxidized isomer of GYM-B at position C-18. In 2015, it was demonstrated that GYM-A, GYM-B, and GYM-C could also be produced by *A. ostenfeldii* [105] (Table 1). The use of phytoplankton net sampling allowed for the identification of GYM-A in Australia (Figure 1), together with *K. selliformis* cells (Moreton Bay, Queensland), and in China (Daya Bay) [45,46]. In terms of toxicity, GYM-A demonstrated high intraperitoneal toxicity in mice (LD_50_ of 80–96 μg kg^−1^), while very low toxicity was reported after oral exposure [106,107]. Moreover, human toxicity is debatable, since no human poisoning could be associated with this toxin.

### 2.9. Brevisulcenals and Brevisulcatic Acids

**Figure 10:**
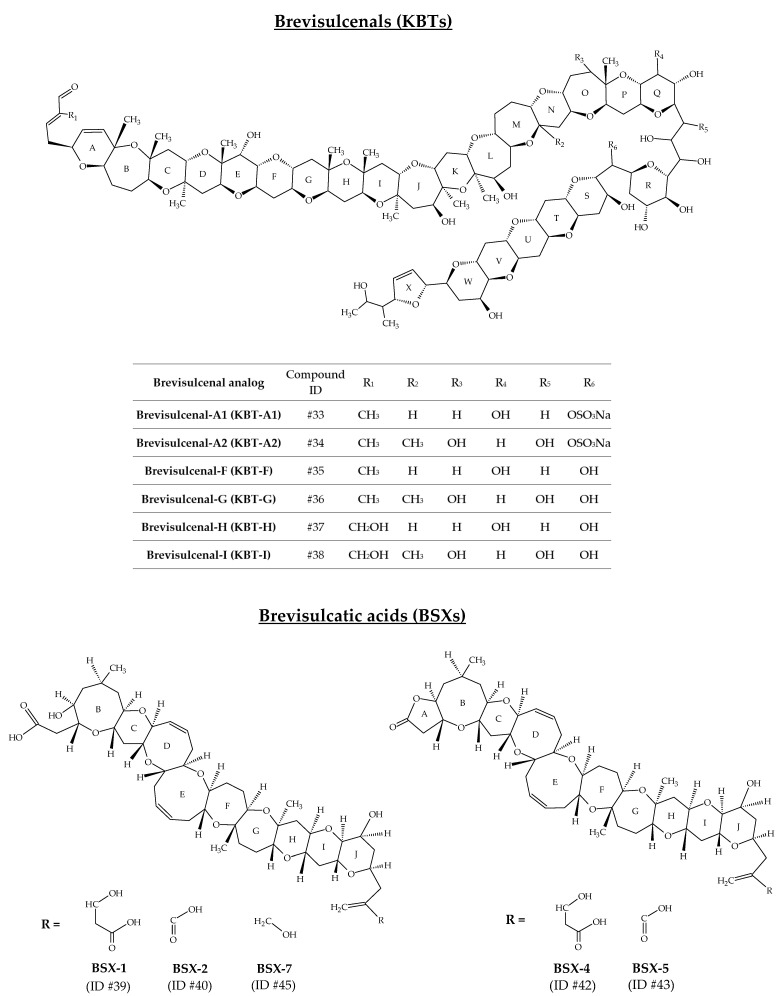
Chemical structures of brevisulcenals (KBTs) and brevisulcatic acids (BSXs).

The toxin complex produced by *K. brevisulcata* was initially designated as Wellington Harbour Toxin (WHT), after the appearance of a bloom in 1998, which devastated all marine life in this New Zealand harbour [108]. This complex is composed of two groups of toxins: brevisulcenals (KBTs for *K. brevisulcata* toxins) and brevisulcatic acids (BSXs) (Figure 10). KBTs are lipophilic polycyclic ethers with a ladder-like structure [109]. Ten analogs are reported (Table 1); however, only six of them have been well characterized to date: KBT-A1 (ID #33), KBT-A2 (ID #34), KBT-F (ID #35), KBT-G (ID #36), KBT-H (ID #37), and KBT-I (ID #38) [75,76,78,82,110] (Figure 10). The mouse i.p. LD_50_ for KBT-F and -G are 0.032 and 0.040 mg/kg bw, respectively. KBT-F and KBT-G are strongly haemolytic, cytotoxic to P388 and neuro-2a cells and highly toxic *i.p*. to mice [82]. Brevisulcatic acids are composed of seven analogs with a common nine cyclic ether backbone. BSX-1 (ID #39), BSX-2 (ID #40), BSX-4 (ID #42) and BSX-5 (ID #43) have known structures and are close to those of BTX-A, especially the ladder arrangement [77,79]. BSX-2, -5, and -7 (ID #45) carry a lactone function. Different side chain substituents make up the various BSX analogs [80]. BSX-3 (ID #41) and BSX-6 (ID #44) were isolated from *K. brevisulcata* cultures by means of ^13^C labelled extracts, combined with the use of liquid chromatography with mass spectrometry detection (LC-MS) acquisitions [77]. Their [M + H]^+^ m/z are 857.5 and 839.5, respectively. However, their molecular formula and chemical structure remain unknown. Harwood et al. [110] suggest that BSX-6 could be an important intermediate in BSX biosynthesis. The mouse *i.p.* LD_50_ for BSX-1 is 3.9 mg/kg, while no deaths were seen in mice injected with BSX-2 at 6.6 mg/kg. The LD_50_ for the lactones BSX-4 and BSX-5 are 1.4 and 1.6 mg/kg, respectively. BSX-4 and -5 are agonists of voltage-gated sodium channels but only weakly haemolytic. Activities in the Neuro-2a cytotoxicity assay were ca 10% of dihydro-brevetoxin-2 and were fully antagonised by saxitoxin [82].

## 3. Biotransformation of Metabolites Produced by *Karenia* spp. in Shellfish

This section will only focus on the metabolism of BTXs and GYMs in shellfish. For both, individual analogs have been reported worldwide (Figure 1), whereas data are lacking for the other groups of metabolites produced by *Karenia* spp. [10,20,21,22,23,24,25,26,27,30,31,32,33,35,36,37,38,39,40,41,42,43,44,45,46,47].

### 3.1. Brevetoxins

#### 3.1.1. Brevetoxins Already Reported from *Karenia* spp.

Several BTXs known to be produced by *Karenia* spp. have also been identified in shellfish (Table 2). Whereas BTX-1 (ID #1) has not been found in shellfish, BTX-2 (ID #8) has occasionally been identified in different species [28,29,30,31]. In fact, both toxins are extensively metabolized, and several metabolic pathways were proposed [31,32]. A study in which eastern oysters (*Crassostrea virginica*) were exposed under controlled conditions to pure BTX-2 revealed that BTX-2 rapidly accumulated and was metabolized into several compounds, including BTX-3 (ID #9), as a reduced form of BTX-2 [28]. Oysters exposed to BTX-3 toxin showed rapid accumulation and significant elimination (about 90%), without any apparent biotransformation, in two weeks. Among the metabolites reported from *K. brevis*, several oxidized, reduced and A-ring lactone hydrolyzed metabolites of BTX-1 and BTX-2 have also been identified in different shellfish species [20,21,30,31,32,33,34]. It is worth noting that the profile of BTX metabolites and their concentrations varied considerably between bivalve mollusk species. For A-type BTXs, oxidized-BTX-1 (ID #4), open A-ring oxidized BTX-1 (ID #6) and open A-ring BTX-7 (ID #7) were reported in hard clams (*mercenaria sp.*) and eastern oysters (*Crassostrea virginica*). For B-type BTXs, BTX-3, BTX-9 (ID #12), and BTX-B5 (ID #13) have been identified in clams (*Mercenaria sp., Macrocallista nimbosa*), cockles (*Austrovenus stutchburyi*), gastropods (*Triplofusus giganteus, Sinistrofulgur sinistrum, Cinctura hunteria, Strombus alatus, Fulguropsis spirata*), mussels (*Perna canaliculus*) and oysters (*Crassostrea gigas, Crassostrea virginica*). BTX-3 was reported at higher level than BTX-2 in shellfish, i.e., in oysters (*Crassostea gigas*) and cockles (*Austrovenus Stutchburyi*) in New Zealand [20,31,33], horse conch (*Triplofusus giganteus*), lightning whelk (*Sinistrofulgur sinistrum*), banded tulip (*Cinctura hunteria*), fighting conch (*Strombus alatus*), pear whelk (*Fulguropsis spirata*), clam (*Mercenaria spp.*) and oyster (*Crassostrea virginica*) in Florida [17,22,111]. BTX-3 and BTX-9 are largely eliminated in oysters after two weeks of depuration [34]. Importantly, open A-ring BTX-1 (ID #5), open A-ring BTX-2 (ID #14), and open A-ring BTX-3 (ID #16), reported from *K. brevis* environmental samples, were not detected. Their extensive metabolism, due to the reactive α,β-unsaturated aldehyde group in their tail region, could explain their absence [35].

#### 3.1.2. Fatty Acid Conjugates of Brevetoxins

**Figure 11:**
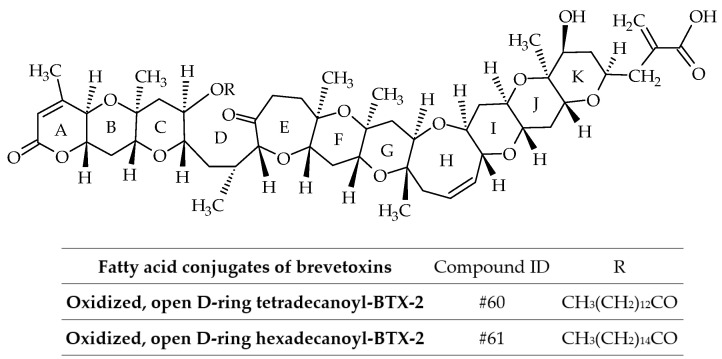
Chemical structures of fatty acid conjugates of brevetoxins (BTXs).

BTX-B3 is not a single compound, but a mixture of two fatty acid conjugates (myristic and palmitic acids) of the open D-ring of BTX-2 with oxidation of the terminal aldehyde (ID #60 and #61) (Figure 11) [36]. Therefore, the use of the term BTX-B3 is discouraged from a chemical perspective (Table 2). These analogs were isolated from greenshell mussels (*Perna canaliculus*) involved in the NSP events of 1992–1993. Interestingly, the mixture of these fatty acid conjugates did not kill mice by intraperitoneal injection at a dose of 300 µg/kg [36]. No further fatty acid conjugates of BTXs (without amino-acid conjugation) were reported.

#### 3.1.3. Amino Acid/Peptide Conjugates of Brevetoxins

**Figure 12:**
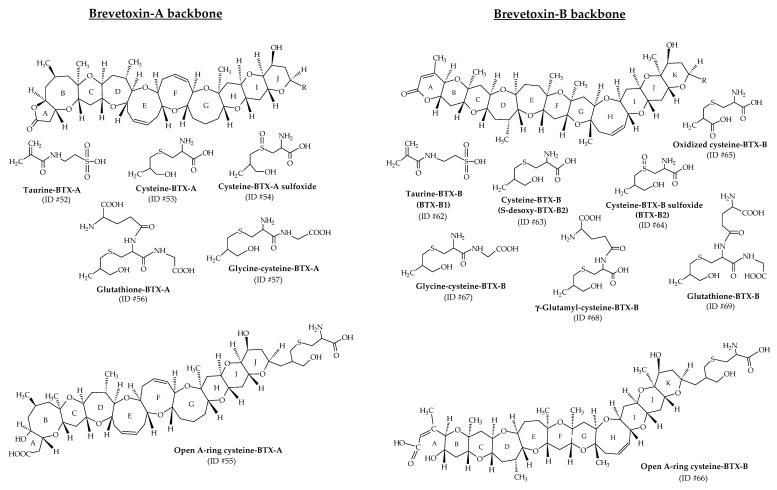
Chemical structures of the amino acid/peptide metabolites of brevetoxins (BTXs) identified from shellfish.

As previously described, taurine-BTX-B (ID #62; also called BTX-B1) and cysteine-BTX-B sulfoxide (ID #64; also called BTX-B2) were the first amino acid conjugates of BTXs identified [37,38], quickly followed by a series of amino acid/peptide conjugates (cysteine, taurine, glycine-cysteine, glutathione, γ-glutamyl-cysteine), among which figure the open A-ring conjugates [22,23,28,34,35,39,48]. Their structures are presented in Figure 12 (ID #52, #53, #54, #55, #56, #57, #62, #63, #64, #65, #66, #67, #68 and #69). Studies from oysters (*Crassostrea virginica*) exposed to *K. brevis* cultures demonstrated the accumulation of several metabolic products like cysteine-BTX-A (ID #53), cysteine-BTX-B (ID #63) and their respective sulfoxides (ID #54 and #64), which were then slowly eliminated [28,34,39]. These metabolites were detectable for up to 6 months in oysters. Additionally, cysteine-BTX-B sulfoxide could also be formed during shellfish extraction or storage of the extracts after sulfoxidation of cysteine-BTX-B [4]. It is important to consider this point to better estimate the actual metabolite content in shellfish in the field. A few years later, after repeated *K. brevis* blooms over a 3-year period in Sarasota Bay (Florida, USA), cysteine-BTX-B and its sulfoxide conjugate were detected in oysters (*Crassostrea virginica*) and were the most abundant and persistent metabolites among those monitored (BTX-3, cysteine and cysteine sulfoxide conjugates of BTX-A and BTX-B) [113]. Six months after experimental exposure to *K. brevis*, both conjugates were still detectable in tissues. The following peptide conjugates were later discovered with BTX-A and BTX-B backbone structures: glycine-cysteine–BTX (m/z 1047 and 1075, with respective ID numbers of #57 and #67), γ-glutamyl-cysteine–BTX (m/z 1147; ID #86), and glutathione–BTX (m/z 1176 and 1204, with respective ID numbers of #56 and #69) [39]. As for BTXs directly produced from *K. brevis*, the lactone group in the A-ring of the conjugate metabolites may open under certain conditions through hydrolysis. Cysteine–BTX-A and cysteine–BTX-B have been identified with their lactone ring opened (ID #55 and #66, respectively) in oysters (*Crassostrea virginica*) [34,35,39]. Binding these amino acids or peptides on the algal toxins decreases lipophilicity and can require method adjustments. The presence of more polar metabolites than algal BTXs has been demonstrated in oysters (*Crassostrea virginica*) that were naturally and experimentally exposed to *K. brevis* [35].

#### 3.1.4. Fatty Acid Derivatives of Brevetoxin Amino acid Conjugates

**Figure 13:**
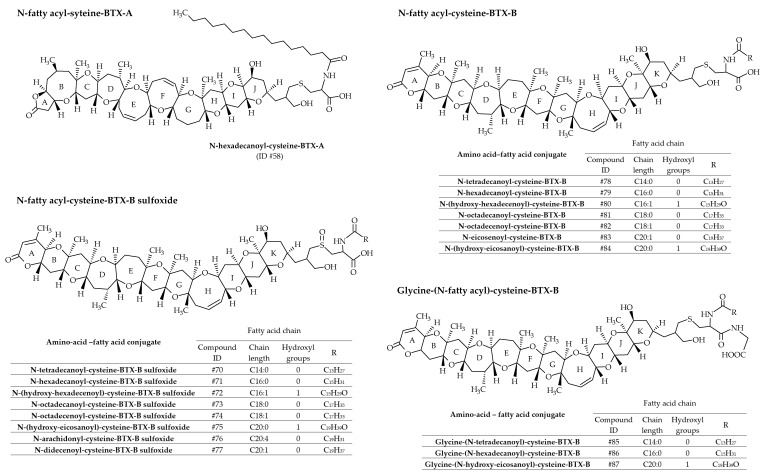
Chemical structures of amino acid-fatty acid conjugates of brevetoxins (BTXs) metabolized in shellfish.

In most cases, fatty acids react with amino acid–BTX conjugates (or peptide-BTX conjugates) through amide linkage to form a series of 19 fatty acid–amino acid–BTX conjugates (ID #58 and #70 to #87 presented in Figure 13). A greater number of amino acid–fatty acid conjugates of BTX-B have been identified compared to BTX-A. BTX-B4, the first isolated, is a mixture of N-tetradecanoyl (N-myristoyl) and N-hexadecanoyl (N-palmitoyl) conjugates with the cysteine sulfoxide moiety of BTX-B2 (ID #70 and #71, respectively) [36,40]. Therefore, as for BTX-B3 (ID #60 and #61), the use of the term BTX-B4 is discouraged. Based on MS/MS fragmentations, Wang et al. [39] reported numerous new analogs, all mentioned in Table 2. Several of these metabolites were confirmed in hard clams (*Mercenaria sp.*) naturally exposed to *K. brevis* blooms [22].

#### 3.1.5. Other Brevetoxin Metabolites

**Figure 14:**
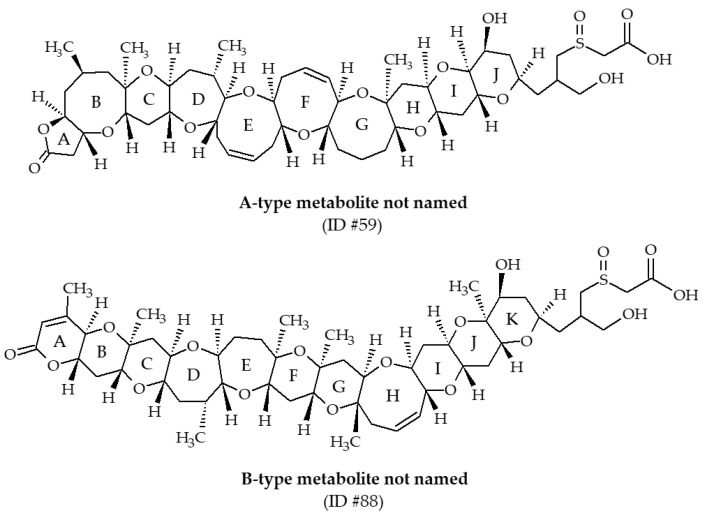
Chemical structures of other brevetoxin (BTX) metabolites identified from shellfish.

Two metabolites, with m/z of 976.5 and 1004.5 (ID #59 and #88, respectively), have been identified from Eastern oysters (*Crassostrea virginica*) collected in the Gulf of Mexico [30,39]. These compounds have not been named. LC-MS/MS analysis revealed the presence of characteristic fragments of A-type and B-type backbone structure of BTXs, allowing Wang et al. [39] to propose structures and fragmentation pathways (Figure 14). In both cases, the side chain possesses sulfoxide and carboxylic acid functions. These metabolites were much more abundant in field-exposed oysters compared with laboratory-exposed oysters. Since 2004, these BTX metabolites have never been reported in the literature.

#### 3.1.6. Biomarkers of Brevetoxin Exposure in Shellfish

The great diversity of physicochemical properties of BTXs complicates the development of efficient multi-toxin analytical methods. Importantly, the products of shellfish metabolism have different lipophilic properties compared to their precursor. The binding of an amino acid or a peptide to the algal toxin contributes to decreased lipophilicity, while binding of a fatty acid increases it. Therefore, the extraction of metabolites with highly variable polarities constitutes a major difficulty. For this reason, different authors emphasized the relevance of use several metabolites as biomarkers of BTX exposure to monitor the toxicity of shellfish following a *K. brevis* bloom [4,18,23]. BTX-3 (ID #9), BTX-B5 (ID #13), taurine-BTX-B (BTX-B1; ID #62), cysteine-BTX-B (S-deoxy-BTX-B2, ID #63), and cysteine-BTX-B sulfoxide (BTX-B2, ID #64) constitute relevant candidates due to their relative persistence. Nevertheless, considering the toxin profile differences between shellfish species, the appropriate biomarkers should be chosen carefully depending on the monitored shellfish species. This high specificity may also complicate the broad application of biomarkers of exposure when different species are targeted. Recently, a strategy was successfully applied to monitor BTX-B in gastropods (*Triplofusus giganteus, Sinistrofulgur sinistrum, Cinctura hunteria, Strombus alatus, Fulguropsis spirata*) and in one species of clams (*Macrocallista nimbosa*) [17]. All gastropod samples were contaminated by BTX-3 (ID #9), BTX-B5 (ID #13), open A-ring BTX-3 (ID #16), taurine-BTX-B (ID #62), cysteine-BTX-B (ID #63) and cysteine-BTX-B sulfoxide (ID #64). Clam samples contained the same metabolites, besides BTX-3 and open A-ring BTX-3.

#### 3.1.7. Monitoring Programs for Brevetoxins in Shellfish

In the United States, brevetoxins widely impact the coasts, especially in the Gulf of Mexico. The closure of production areas is based on *Karenia* spp. cell count or toxin analysis in shellfish tissues. A maximum limit of 20 mouse units per 100 g of tissue is applied using the mouse bioassay (MBA) and, with certain limitations, the enzyme linked immunosorbent assay (ELISA), as described in the National Shellfish Sanitation Program [114]. The re-opening of production areas is based on toxin analysis. With these analytical tools, the identity of the analogs remains unknown. In Australia, production area closures have different strategies depending on the federal states. MBA is implemented in Victoria and New South Wales; however, an LC-MS/MS analysis is also carried out in New South Wales [115,116]. BTX-2 (ID #8), BTX-3 (ID #9), BTX-B5 (ID #13), taurine-BTX-B (BTX-B1; ID #62), cysteine-BTX-B (S-deoxy-BTX-B2; ID #63), and cysteine-BTX-B sulfoxide (BTX-B2; ID #64) are the BTX analogs sought [117]. In New Zealand, BTXs are regulated (maximum permissible level of 0.8 mg BTX-2 equivalent per kg of shellfish meat) and the reference method is based on LC-MS [118]. In France, BTX-2 and BTX-3 have been monitored since 2018 [10] as part of a program on emerging toxins in marine shellfish (EMERGTOX). Recently, a working group was set up by the French Agency for Food, Environmental and Occupational Health and Safety (ANSES) to prevent health risks associated with the consumption of shellfish contaminated with BTXs [11,119]. A guidance level of 180 µg BTX-3 eq./kg shellfish meat was established, considering a protective default portion size of 400 g shellfish meat. A monitoring strategy has also been proposed and is divided into three parts [119]. First, the implementation of an ELISA test for the screening of B-type brevetoxin metabolites in shellfish flesh. This approach considers brevetoxins not quantifiable by LC-MS/MS due to the lack of commercial standards. Second, the targeted physicochemical analysis of brevetoxins for which standards are available with an LC-MS/MS method developed for a wide range of polarity. Based on available reference materials and toxicity data, a list of analogs to seek first was established and includes: BTX-1 (ID #1), BTX-2 (ID #8), BTX-3 (ID #9), oxidized open D-ring tetradecanoyl-BTX-2 (BTX-B3; ID #60), oxidized open D-ring hexadecanoyl-BTX-2 (BTX-B3; ID #61), taurine-BTX-B (BTX B1; ID #62), cysteine-BTX-B (S-desoxy-BTX-B2; ID #63), cysteine-BTX-B sulfoxide (BTX-B2; ID #64), N-tetradecanoyl-cysteine-BTX-B sulfoxide (N-myristoyl-BTX-B2; ID #70), and N-hexadecanoyl-cysteine-BTX-B sulfoxide (N-palmitoyl-BTX-B2; ID #71). Lastly, the implementation of a non-targeted analysis using liquid chromatography-high resolution mass spectrometry (LC-HRMS) has been suggested to screen a large number of compounds and identify possible new BTX analogs or degradation products.

### 3.2. Gymnodimines (GYMs)

**Figure 15:**
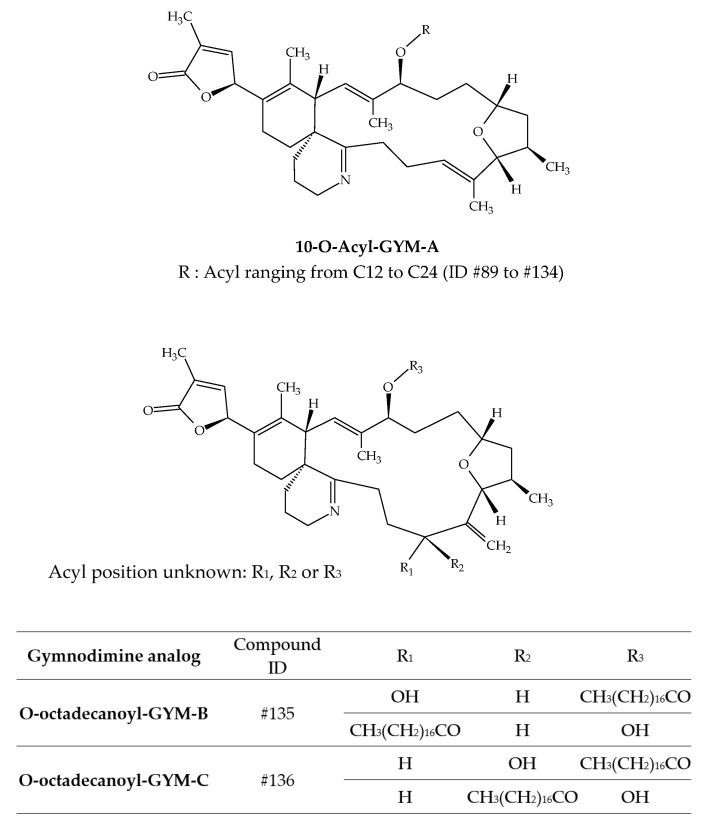
Chemical structures of fatty acid conjugates of gymnodimines (GYMs).

Several studies have demonstrated the accumulation of gymnodimines (GYMs) in different shellfish species (Table 3). The discovery of GYM-A (ID #30) from New Zealand oysters (*Tiostrea chilensis*) in 1995 immediately highlighted this capacity [24]. A few years later, several batches of clams harvested in Tunisia were neurotoxic to mice by *i.p.* [27]. Analytical investigations allowed for the unequivocal identification of GYM-A. Several studies on Tunisian samples followed [25,26]. In 2012, GYM-A, GYM-B (ID #31) and GYM-C (ID #32) were detected in the same shellfish species collected in the Gulf of Gabes, along with the presence of *K. selliformis* [25]. Other studies were carried out in South Africa, Australia, and China (Figure 1) and allowed for the detection of GYM-A in oysters, gastropods, mussels, donax and pen shells [43,44,45,46,47]. In 2013, numerous gymnodimine fatty acid conjugates were identified from grooved carpet shells (*Ruditapes decussatus*) collected in Tunisia (Gulf of Gabes) [42]. In total, 46 fatty acid conjugates of GYM-A (ID #89 to ID #134) have been reported, with acyl carbon chain lengths comprised between C12:0 and C24:6 (Table 3). The presence of a series of GYM-B and GYM-C fatty acid conjugates was also established, with esters of 16, 18, 20 and 22 carbon chain lengths being the most abundant. However, only the 18:0 esters of GYM-B and GYM-C were specifically identified, with respective ID number of #135 and # ID136 (Figure 15). Some of the GYM-A fatty acids conjugates were also identified in shellfish collected in the South China Sea [41]. Clam (*Antigona lamellaris*) and pen shell (*Atrina pectinata*) profiles were different. In clams, the 18:0 ester was the most abundant, followed by the 20:1 ester, whereas the 20:1, 18:0 and 22:2 esters dominated the acyl ester profile in pen shell samples. These authors also experimentally exposed mussels (*Mytilus galloprovincialis*) to *K. selliformis* for 96 h. In all, 28 fatty acids esters were detected with acyl carbon chain lengths ranging between C14:0 and C24:6. Interestingly, GYM-A was detected in oysters (*Crassostrea sp.*) and gastropods (*Batillaria zonalis*), but no GYM-A esters were observed in these species. Further investigations are required to obtain better knowledge of GYM toxicity. However, considering the significant presence of GYM acyl esters, more occurrence data are required to conduct a risk assessment at a later stage.

## 4. Accumulation and Biotransformation of Metabolites Produced by *Karenia* spp. in Other Marine Organisms

Blooms of *K. brevis* are commonly associated with massive mortality for marine organisms. Massive death episodes of fish (prey fishes, sharks, rays), mammals (dolphins, manatees) or shorebirds, all occurring in Florida between 1999 and 2006, were associated with *K. brevis* blooms and the presence of BTXs [5,49,50,120]. Data regarding the accumulation and biotransformation of the other groups of toxins produced by *Karenia* spp. are lacking. In several studies, the implementation of biochemical methods of analysis, such as enzyme-linked immunosorbent assay (ELISA), radioimmunoassay (RIA), or receptor-binding assay (RBA), made it possible to measure high levels of BTX-like toxins in animal tissues, but did not enable identification of the specific nature of the BTX analogs. In a few studies conducted on samples collected in the Gulf of Mexico (Figure 1), LC-MS and LC-MS/MS analyses were implemented to obtain this information [2,48,49,50].

In fish, BTX-3 (ID #9) was identified in *Lagodon rhomboides* and *Leiostomus xanthurus*, whereas open A-ring BTX-3 (ID #16) and open A-ring BTX-7 (ID #7), not reported in shellfish to date, were also reported [48]. Among the amino-acid conjugates, cysteine-BTX-A (ID #53), cysteine BTX-A sulfoxide (ID #54), cysteine-BTX-B (ID #61) and cysteine-BTX-B sulfoxide (ID #62) were also identified in these fish species. From 301 live or dead sharks and rays from Florida, Flewelling at al. [49] studied the accumulation of BTXs. Some shark embryos were also included. For dead animals, the cause of their death was unknown but sometimes occurred during red-tide episodes of *K. brevis* blooms. Among these samples, a subset of liver and gill tissues were selected, and several metabolites were sought through LC-MS analysis (BTX-1, BTX-2, BTX-3, BTX-6, BTX-7, BTX-9, BTX-10, cysteine-BTX-B, cysteine-BTX-B sulfoxide and brevenal with respective ID numbers #1, #8, #9, #11, #2, #12, #3, #61, #62, #20). BTXs were detected in 26 of the 27 selected extracts. BTX-3 was quantified in most of the liver and gill tissues (n = 21), whereas BTX-2 was only quantified in gills of two sharks. Cysteine-BTX-B and its sulfoxide were detected in 18 and 9 extracts of gills or livers, respectively.

After major dolphin (*Tursiops truncatus*) mortality events between 1999 and 2006 in Florida, BTXs and domoic acid (amnesic toxin produced by the *Pseudo-nitzschia* diatom) were sought in dead animals [50]. Of the 105 animals collected in 2004, 100% of the tested animals were positive for BTXs and 89% for domoic acid. Moreover, dolphin stomach contents frequently consisted of BTX-contaminated menhaden (*Brevoortia sp.*). For the period 2005–2006, 93% of the 90 dolphins were positive for BTXs, whereas domoic acid was not detected in these animals. Among the samples analyzed by LC-MS, BTX-3 (ID #9) was the predominant toxin in the four stomach contents tested (fishes partially digested) and was also detected in 10 of the 12 livers tested. Two peaks co-eluting with BTX-2 were not attributed to any known toxin.

An experimental study on shrimps (*Litopenaeus vannamei*) demonstrated that these marine organisms could represent a vector of BTXs [112]. The authors found by LC-MS levels of 80 and 90 µg eq BTX-2/kg in the digestive glands and muscles, respectively. It should be noted that this contamination started to appear in the digestive gland and the muscle after 30 and 45 days of exposure, respectively, and with relatively low cellular concentrations of *K. brevis* (10^3^ to 10^6^ cells/L). Macrobenthic invertebrates (including polychaetes and crustaceans belonging to amphipods and isopods) collected during a *K. brevis* bloom in Florida also demonstrated their capacity to accumulate BTXs [2]. However, the identity of the analogs was not determined due to the analytical tool implemented for the analysis (ELISA test).

## 5. Conclusions

In this review, we summarized and gathered the knowledge reported in the literature over recent decades of over 130 potentially toxic metabolites reported from *Karenia* spp. or metabolized by marine organisms. The structures and certain properties of these metabolites are presented. This information could constitute an interesting basis in view of library-building, particularly for physicochemical analysis using liquid chromatography combined with high-resolution mass spectrometry (LC-HRMS). Such an approach could be an interesting tool to simultaneously screen the different analogs mentioned here, and could also allow us to discover new analogs. The accumulation and biotransformation by marine organisms of the metabolites produced by *Karenia* spp. was also assessed in depth for BTXs and GYMs. We point out the need for more information on BTXs in marine organisms other than shellfish. Occurrence data would also be valuable for several groups of potentially toxic metabolites discovered in the last two decades. These data, combined with additional toxicology assays, could be used to carry out a risk assessment. Lastly, we compiled all the names reported for metabolites in the existing literature. Among the different nomenclatures, we tried to select the terms that allowed us to obtain a better overall consistency, hoping that this work will be useful to harmonize practices in the future.

## Figures and Tables

**Table 1:** Physicochemical properties of brevetoxins (BTXs) and other potentially toxic metabolites produced by *Karenia* species.

Group	Compound Identification Number (ID)	Metabolite	Other Existing Names	Molecular Formula	Monoisotopic Mass (Da)	LogP ^1^	Species	First Identification/Structural Elucidation References
A-type brevetoxins (BTX-A)	#1	BTX-1	PbTx-1; GB-1; T46, BTX-A	C_49_H_70_O_13_	866.5	5.8	*K. brevis*	[60]
	#2	BTX-7	PbTx-7; GB-7; Aldehyde-reduced PbTx-1	C_49_H_72_O_13_	868.5	5.7	*K. brevis*	[60]
	#3	BTX-10	/	C_49_H_74_O_13_	870.5	5.5	*K. brevis*	[13,61] ^2^
	#4	Oxidized BTX-1	Oxidized PbTx-1 PbTx-1-carboxylic acid	C_49_H_70_O_14_	882.5	/	*K. brevis*	[35] ^3^
	#5	Open A-ring BTX-1	Open-ring PbTx-1	C_49_H_72_O_14_	884.5	/	*K. brevis*	[35] ^3^
	#6	Open A-ring, oxidized BTX-1	Open-ring, oxidized PbTx-1; PbTx-1-open ring-carboxylic acid	C_49_H_72_O_15_	900.5	/	*K. brevis*	[35] ^3^
	#7	Open A-ring BTX-7	Open-ring PbTx-7	C_49_H_74_O_14_	886.5	/	*K. brevis*	[35] ^3^
B-type brevetoxins (BTX-B)	#8	BTX-2	PbTx-2; T47; GB-2, T34, BTX-B	C_50_H_70_O_14_	894.5	6.4	*K. brevis*	[62,63,64]
	#9	BTX-3	PbTx-3; GB-3; T17; Dihydro-BTX-B; Aldehyde-reduced PbTx-2	C_50_H_72_O_14_	896.5	6.4	*K. brevis*	[65,66]
	#10	BTX-5	PbTx-5; GB-5; acetylated PbTx-2	C_52_H_72_O_15_	936.5	7.1	*K. brevis*	[67]
	#11	BTX-6	PbTx-6; GB-6; 27,28 epoxyde of PbTx-2	C_50_H_70_O_15_	910.5	7.3	*K. brevis*	[67]
	#12	BTX-9	PbTx-9; α-methylene reduced PbTx-3	C_50_H_74_O_14_	898.5	6.3	*K. brevis*	[13,14,61]
	#13	BTX-B5	Oxidized BTX-2; Oxidized PbTx-2	C_50_H_70_O_15_	910.5	/	*K. brevis*	[32,33,35]
	#14	Open A-ring BTX-2	Open A-ring PbTx-2	C_50_H_72_O_15_	912.5	/	*K. brevis*	[35] ^3^
	#15	Open A-ring, oxidized BTX-2	Open A-ring, Oxidized PbTx-2; Open A-ring BTX-B5; PbTx-2-open ring-carboxylic acid	C_50_H_72_O_16_	928.5	/	*K. brevis*	[35] ^3^
	#16	Open A-ring BTX-3	Open A-ring PbTx-3	C_50_H_74_O_15_	914.5	/	*K. brevis*	[35] ^3^
Hemi- Brevetoxins (Hemi-BTXs)	#17	Hemi-BTX-A	GB-M	/	/	/	*K. brevis*	[68,69]
	#18	Hemi-BTX-B	GB-N	C_28_H_42_O_7_	490.3	3.5	*K. brevis*	[69]
	#19	Hemi-BTX-C	GB-4	/	/	/	*K. brevis*	[69]
Brevenals	#20	Brevenal	/	C_39_H_60_O_8_	656.4	6.9	*K. brevis*	[70]
	#21	Dimethyl acetal brevenal	/	C_41_H_66_O_9_	702.5	/	*K. brevis*	[70]
Brevisamide	#22	Brevisamide	/	C_18_H_29_NO_4_	323.2	/	*K. brevis*	[71]
Brevisin	#23	Brevisin	/	C_39_H_62_O_11_	706.4	3.6	*K. brevis*	[72]
Tamulamides (Tams)	#24	Tam-A	/	C_35_H_45_NO_10_	639.3	1.0	*K. brevis*	[73]
	#25	Tam-B	/	C_34_H_43_NO_10_	625.3	0.7	*K. brevis*	[73]
Gymnocins	#26	Gymnocin-A	/	C_55_H_80_O_18_	1028.5	3.3	*K. mikimotoi*	[56]
	#27	Gymnocin-A carboxylic acid	/	C_55_H_80_O_19_	1044.5	3.6	*K. mikimotoi*	[58]
	#28	Gymnocin-A2	/	C_55_H_80_O_18_	1028.5	/	*K. mikimotoi*	[58]
	#29	Gymnocin-B	/	C_62_H_92_O_20_	1156.6	5.0	*K. mikimotoi*	[57]
Gymnodimines (GYMs)	#30	GYM-A	/	C_32_H_45_NO_4_	509.4	6.4	*K. mikimotoi K. selliformis*	[24]
	#31	GYM-B	/	C_32_H_45_NO_5_	523.3	5.1	*K. selliformis*	[74]
	#32	GYM-C	GYM-B isomer	C_32_H_45_NO_5_	523.3	/	*K. selliformis*	[59]
Brevisucenals (KBTs)	#33	KBT-A1 (sodium salt)	KBT-F sulfate ester	C_107_H_159_O_41_SNa	2155.0	/	*K. brevisulcata*	[75]
	#34	KBT-A2 (sodium salt)	KBT-G sulfate ester	C1_08_H_161_O_42_SNa	2185.0	/	*K. brevisulcata*	[75]
	#35	KBT-F	/	C_107_H_160_O_38_	2053.1	7.9	*K. brevisulcata*	[76,77]
	#36	KBT-G	/	C_108_H_162_O_39_	2083.1	/	*K. brevisulcata*	[77,78]
	#37	KBT-H	/	C_107_H_160_O_39_	2069.1	/	*K. brevisulcata*	[78]
	#38	KBT-I	/	C_108_H_162_O_40_	2099.1	/	*K. brevisulcata*	[78]
Brevisulcatic acids (BSXs)	#39	BSX-1	/	C_49_H_72_O_16_	916.5	3.6	*K. brevisulcata*	[77,79]
	#40	BSX-2	/	C_47_H_68_O_15_	872,5	/	*K. brevisulcata*	[77,80]
	#41	BSX-3	/	/	856.5	/	*K. brevisulcata*	[77] ^4^
	#42	BSX-4	/	C_49_H_70_O_15_	898.5	3.8	*K. brevisulcata*	[68,79]
	#43	BSX-5	/	C_47_H_66_O_14_	854.4	/	*K. brevisulcata*	[77,80]
	#44	BSX-6	Lactone derivative of BSX-3	/	838.5	/	*K. brevisulcata*	[77] ^4^
	#45	BSX-7	/	C_47_H_70_O_14_	858.5	/	*K. brevisulcata*	[80]

^1^ LogP predicted from the chemical structure of molecules using the ACD/Labs platform. Octanol/water partition coefficients were predicted using algorithms. ^2^ Synthesized toxin, unidentified in cultures to date, but postulated to occur naturally, based on structural correlation with BTX-9 and its presence in stationary cultures. ^3^ Analogs postulated after thorough study of LC-MS/MS fragmentations. ^4^ Structure not elucidated.

**Table 2:** Physicochemical characteristics of brevetoxin (BTX) metabolites reported in marine organisms.

Group	Compound Identification Number (ID)	Metabolite	Other Existing Names	Molecular Formula	Monoisotopic Mass (Da)	LogP ^1^	Sample	References
A-type BTXs	#4	Oxidized BTX-1	Oxidized PbTx-1; PbTx-1-carboxylic acid	C_49_H_70_O_14_	882.5	/	Clams (*mercenaria sp.*)	[22]
	#6	Open A-ring, oxidized BTX-1	Open A-ring of oxidized PbTx-1; A-type opened A-ring derivative	C_49_H_72_O_15_	900.5	/	Clams (*mercenaria sp.*); Oysters (*Crassostrea virginica*)	[22,35]
	#7	Open A-ring BTX-7	Open A-ring PbTx-7	C_49_H_74_O_14_	886.5	/	Oysters (*Crassostrea virginica*); Pinfish (*Lagodon rhomboides*); Spot (*Leiostomus xanthurus*)	[35,39,48]
	#52	Taurine-BTX-A	N-taurine conjugate of oxidized BTX-1	C_51_H_75_NO_16_S	989.5	/	Clams (*mercenaria sp.*)	[22]
	#53	Cysteine–BTX-A	Cysteine–PbTx-A; Cysteine-PbTx-1; Cysteine-BTX-1	C_52_H_79_NO_15_S	989.5	/	Clams (*mercenaria sp.*); Oysters (*Crassostrea virginica*); Pinfish (*Lagodon rhomboides*); Spot (*Leiostomus xanthurus*)	[23,35,39,48]
	#54	Cysteine–BTX-A sulfoxide	Cysteine–PbTx-A sulfoxide; Sulfoxide cysteine conjugate of PbTx-1	C_52_H_79_NO_16_S	1005.5	/	Clams (*mercenaria sp.*); Oysters (*Crassostrea virginica*); Pinfish (*Lagodon rhomboides*); Spot (*Leiostomus xanthurus*)	[23,35,39,48]
	#55	Open A-ring cysteine-BTX-A	Open A-ring cysteine–PbTx-A	C_52_H_81_NO_16_S	1007.5	/	Clams (*mercenaria sp.*); Oysters (*Crassostrea virginica*)	[22,34,35,39]
	#56	Glutathione-BTX-A	Glutathione-PbTx-A	C_59_H_89_N_3_O_19_S	1175.6	/	Oysters (*Crassostrea virginica*)	[23,34,39]
	#57	Glycine-cysteine-BTX-A	Glycine-cysteine-PbTx-A	C_54_H_82_N_2_O_16_S	1046.5	/	Clams (*mercenaria sp.*); Oysters (*Crassostrea virginica*)	[23,39]
	#58	N-hexadecanoyl-cysteine–BTX-A	N-hexadecanoyl-cysteine–PbTx-A	C_68_H_109_NO_16_S	1227.7	/	Oysters (*Crassostrea virginica*)	[39]
	#59	Metabolite not named	/	C_51_H_76_O_16_S	976.5	/	Oysters (*Crassostrea virginica*)	[39]
B-type BTXs	#8	BTX-2	PbTx-2; T47; GB-2; T34; BTX-B	C_50_H_70_O_14_	894.5	6.2	Cockles (*Austrovenus stutchburyi);* Mussels (*Perna canaliculus*); Oysters (*Crassostrea virginica, Crassostrea gigas*); Sharks (*Rhizoprionodon terraenovae, Sphyrna tiburo*); Shrimps (*Litopenaeus vannamei*)	[10,21,30,31,32,49,64,112]
	#9	BTX-3	PbTx-3; GB-3; T17; dihydro-BTX-B; aldehyde-reduced PbTx-2	C_50_H_72_O_14_	896.5	6.4	Bottlenose dolphins (*Tursiops Truncatus*); Clams (*Mercenaria sp.*); Cockles (*Austrovenus stutchburyi);* Gastropods (*Triplofusus giganteus, Sinistrofulgur sinistrum, Cinctura hunteria, Strombus alatus, Fulguropsis spirata*); Mussels (*Perna canaliculus*); Oysters (*Crassostrea gigas, Crassostrea virginica*); Pinfish (*Lagodon rhomboides*); Rays (*Dasyatis sabina*); Sharks (*Carcharhinus limbatus, Rhizoprionodon terraenovae, Sphyrna tiburo*); Spot (*Leiostomus xanthurus*)	[10,17,20,21,22,31,32,33,48,49,50,66]
	#16	Open A-ring BTX-3	Open-ring PbTx-3	C_50_H_74_O_15_	914.5	/	Gastropods (*Triplofusus giganteus, Sinistrofulgur sinistrum, Cinctura hunteria, Strombus alatus, Fulguropsis spirata*); Oysters (*Crassostrea virginica*); Pinfish (*Lagodon rhomboides*); Spot (*Leiostomus xanthurus*)	[17,35,48]
	#12	BTX-9	Reduced-BTX-2	C_50_H_74_O_14_	898.5	6.3	Oysters (*Crassostrea virginica*)	[34,39]
	#13	BTX-B5	Oxidized BTX-2; Oxidized PbTx-2; PbTx-2-carboxylic acid	C_50_H_70_O_15_	910.5	6.7	Clams (*Mercenaria sp., Macrocallista nimbosa);* Cockles (*Austrovenus stutchburyi*); Gastropods (*Triplofusus giganteus, Sinistrofulgur sinistrum, Cinctura hunteria, Strombus alatus, Fulguropsis spirata*); Mussels (*Perna canaliculus*); Oysters (*Crassostrea gigas*, *Crassostrea virginica*)	[17,22,23,31,32,33,39]
	#15	Open A-ring, oxidized BTX-2	Open ring, oxidized PbTx-2; Open A-ring BTX-B5; PbTx-2-open ring-carboxylic acid	C_50_H_72_O_16_	928.5	/	Clams (*mercenaria sp*.); Oysters (*Crassostrea virginica*)	[22,23,35]
	#60	Oxidized, open D-ring tetradecanoyl-BTX-2	BTX-B3; Oxidized, open D-ring myristoyl-BTX-2	C_64_H_96_O_17_	1136.7	/	Mussels (*Perna canaliculus*)	[36]
	#61	Oxidized, open D-ring hexadecanoyl-BTX-2	BTX-B3; Oxidized, open D-ring palmitoyl-BTX-2	C_66_H_100_O_17_	1164.7	/	Mussels (*Perna canaliculus*)	[36]
	#62	Taurine-BTX-B	BTX-B1; N-taurine conjugate of oxidized PbTx-2; N-taurine conjugate of BTX-B5	C_52_H_75_NO_17_S ^2^	1017.5 ^2^	/	Clams (*Mercenaria sp.; Macrocallista nimbosa*); Cockles (*Austrovenus stutchburyi);* Gastropods (*Triplofusus giganteus, Sinistrofulgur sinistrum, Cinctura hunteria, Strombus alatus, Fulguropsis spirata*); Mussels (*Perna canaliculus*); Oysters (*Crassostrea gigas*)	[17,20,22,23,31,32,33,37]
	#63	Cysteine-BTX-B	S-desoxy-BTX-B2; S-deoxy-BTX-B2; Cysteine-BTX-B; Cysteine-PbTx; Cysteine-PbTx-B; Cysteine conjugate of PbTx-2	C_53_H_79_NO_16_S	1017.5	/	Clams (*Mercenaria sp.*; *Macrocallista nimbosa*); Gastropods (*Triplofusus giganteus, Sinistrofulgur sinistrum, Cinctura hunteria, Strombus alatus, Fulguropsis spirata*); Oysters (*Crassostrea virginica*); Pinfish (*Lagodon rhomboides*); Sharks (*Carcharhinus limbatus, Rhizoprionodon terraenovae, Sphyrna tiburo*); Spot (*Leiostomus xanthurus*); Rays (*Dasyatis sabina*)	[17,22,23,28,34,35,48,49]
	#64	Cysteine-BTX-B sulfoxide	BTX-B2; Cysteine-PbTx-B sulfoxide; Cysteine-PbTx sulfoxide; Oxidised-S-desoxy-BTX-B2	C_53_H_79_NO_17_S	1033.5	4.3	Clams (*Mercenaria sp., Macrocallista nimbosa*); Gastropods (*Triplofusus giganteus, Sinistrofulgur sinistrum, Cinctura hunteria, Strombus alatus, Fulguropsis spirata*); Mussels (*Perna canaliculus*); Oysters (*Crassostrea virginica*); Pinfish (*Lagodon rhomboides*); Sharks (*Carcharhinus limbatus, Rhizoprionodon terraenovae, Sphyrna tiburo*); Spot (*Leiostomus xanthurus*); Rays (*Dasyatis sabina*)	[17,22,23,28,34,35,38,48,49]
	#65	Oxidized cysteine-BTX-2	Cysteine conjugate of oxidized PbTx-2	C_53_H_77_NO_17_S	1031.5	/	Oysters (*Crassostrea virginica*)	[39]
	#66	Open A-ring cysteine-BTX-B	Open A-ring S-desoxy-BTX-B2; Open A-ring cysteine–PbTx-B; Open A-ring cysteine–PbTx-2	C_53_H_81_NO_17_S	1035.5	/	Clams (*mercenaria sp.*); Oysters (*Crassostrea virginica*)	[22,34,35,39]
	#67	Glycine-cysteine-BTX-B	Glycine-cysteine-PbTx-B	C_55_H_82_N_2_O_17_S	1074.5	/	Clams (*mercenaria sp.*); Oysters (*Crassostrea virginica*)	[22,34,39]
	#68	γ-glutamyl-cysteine-BTX-B	γ-glutamyl-cysteine-PbTx-B	C_58_H_86_N_2_O_19_S	1146.6	/	Clams (*mercenaria sp.*); Oysters (*Crassostrea virginica*)	[22,34,39]
	#69	Glutathione-BTX-B	Glutathione-PbTx-B	C_60_H_89_N_3_O_20_S	1203.6	/	Clams (*mercenaria sp.*); Oysters (*Crassostrea virginica*)	[22,34,39]
	#70	N-tetradecanoyl-cysteine-BTX-B sulfoxide	BTX-B4; N-tetradecanoyl-BTX-B2; N-myristoyl-BTX-B2; N-myristoyl-cysteine- PbTx-B sulfoxide	C_67_H_105_NO_18_S	1243.7	11.3	Mussels (*Perna canaliculus*); Oysters (*Crassostrea virginica*)	[39,40]
	#71	N-hexadecanoyl-cysteine-BTX-B sulfoxide	BTX-B4; N-hexadecanoyl-BTX-B2; N-palmitoyl-BTX-B2; N-hexadecanoyl-cysteine–PbTx-B sulfoxide	C_69_H_109_NO_18_S	1271.7	12.3	Clams (*mercenaria sp.*); Mussels (*Perna canaliculus*); Oysters (*Crassostrea virginica*)	[22,39,40]
	#72	N- (hydroxy-hexadecenoyl)-cysteine-BTX-B sulfoxide	N- (hydroxy-hexadecenoyl)-BTX-B2	C_69_H_107_NO_19_S	1285.7	/	Clams (*mercenaria sp.*)	[22]
	#73	N-octadecanoyl-cysteine-BTX-B sulfoxide	N-octadecanoyl-BTX-B2	C_71_H_113_NO_18_S	1299.8	/	Clams (*mercenaria sp.*)	[22]
	#74	N-octadecenoyl-cysteine-BTX-B sulfoxide	N-octadecenoyl-BTX-B2	C_71_H_111_NO_18_S	1297.8	/	Clams (*mercenaria sp.*)	[22]
	#75	N- (hydroxy-eicosanoyl)-cysteine-BTX-B sulfoxide	N- (hydroxy-eicosanoyl)-BTX-B2	C_73_H_117_NO_19_S	1343.8	/	Clams (*mercenaria sp.*)	[22]
	#76	N-arachidonyl-cysteine-BTX-B sulfoxide	N-arachidonyl-BTX-B2	C_73_H_109_NO_18_S	1319.7	/	Clams (*mercenaria sp.*)	[22]
	#77	N-didecenoyl-cysteine-BTX-B sulfoxide	N-didecenoyl-BTX-B2	C_73_H_115_NO_18_S	1325.8	/	Clams (*mercenaria sp.*)	[22]
	#78	N-tetradecanoyl-cysteine-BTX-B	N-tetradecanoyl-cysteine–PbTx-B	C_67_H_105_NO_18_S	1243.7	/	Oysters (*Crassostrea virginica*)	[39]
	#79	N-hexadecanoyl-cysteine-BTX-B	N-hexadecanoyl-cysteine–PbTx-B; N-hexadecanoyl-S-deoxy-BTX-B2	C_69_H_109_NO_17_S	1255.7	/	Clams (*mercenaria sp.*); Oysters (*Crassostrea virginica*)	[22,39]
	#80	N-(hydroxy-hexadecenoyl)-cysteine-BTX-B	N-(hydroxy-hexadecenoyl)-S-deoxy-BTX-B2	C_69_H_107_NO_18_S	1269.7	/	Clams (*mercenaria sp.*)	[22]
	#81	N-octadecanoyl-cysteine-BTX-B	N-octadecanoyl-cysteine–PbTx-B; N-octadecanoyl-S-deoxy-BTX-B2	C_71_H_113_NO_17_S	1283.8	/	Clams (*mercenaria sp.*); Oysters (*Crassostrea virginica*)	[22,39]
	#82	N-octadecenoyl-cysteine-BTX-B	N-octadecenoyl-cysteine–PbTx-B	C_71_H_111_NO_17_S	1281.8	/	Oysters (*Crassostrea virginica*)	[39]
	#83	N-eicosenoyl-cysteine-BTX-B	N-eicosenoyl-cysteine–PbTx-B	C_73_H_115_NO_17_S	1309.8	/	Oysters (*Crassostrea virginica*)	[39]
	#84	N- (hydroxy-eicosanoyl)-cysteine-BTX-B	N-(hydroxy-eicosanoyl)-cysteine–PbTx-B; N-(hydroxy-eicosanoyl)-S-deoxy-BTX-B2	C_73_H_117_NO_18_S	1327.8	/	Clams (*mercenaria sp.*); Oysters (*Crassostrea virginica*)	[22,39]
	#85	Glycine-(N-tetradecanoyl)-cysteine-BTX-B	Glycine-(N-tetradecanoyl-cysteine)–PbTx-B	C_69_H_108_N_2_O_18_S	1284.7	/	Oysters (*Crassostrea virginica*)	[39]
	#86	Glycine-(N-hexadecanoyl)-cysteine-BTX-B	Glycine-(N-hexadecanoyl-cysteine)– PbTx-B	C_71_H_112_N_2_O_18_S	1312.8	/	Oysters (*Crassostrea virginica*)	[39]
	#87	Glycine–(N-hydroxy-eicosanoyl)-cysteine-BTX-B	Glycine–(N-hydroxy-eicosanoyl)-cysteine–PbTx-B	C_75_H_120_N_2_O_19_S	1384.8	/	Oysters (*Crassostrea virginica*)	[39]
	#88	Metabolite not named	/	C_52_H_76_O_17_S	1004.5	/	Oysters (*Crassostrea virginica*)	[30,39]

^1^ LogP predicted from the chemical structure of molecules using the ACD/Labs platform. Octanol/water partition coefficient are predicted using algorithms. ^2^ Sometimes refers to the sodium salt of taurine-BTX-B with a molecular formula of C_52_H_74_NO_17_SNa and a monoisotopic mass of 1039.5 Da [20,37].

**Table 3:** Physicochemical characteristics of gymnodimine (GYM) metabolites reported in shellfish (only gymnodimine analogs produced from *K. selliformis*).

Gymnodimine (GYM) Metabolite	Compound Identification Number (ID)	Carboxyl Group of the Ester (Carbon:Unsaturation)	Molecular Formula	Monoisotopic Mass (Da)	LogP ^1^	Sample	References
GYM-A	#30	/	C_32_H_45_NO_4_	507.3	6.4	Clams (*Antigona lamella*, *Ruditapes decussatus*); Gastropods (*Batillaria zonalis*); Oysters (*Crassostrea angulata, Crassostrea ariakensis, Crassostrea giga, Dendostrea crenulifrea*, *Saccostrea glomerata*, *Tiostrea chilensis*); Mussels (*Choromytilus meridionalis, Mytilus galloprovincialis, Modiolus proclivis*); Pipis (*Donax deltoides*); Pen shells (*Atrina pectinata*);	[24,25,26,27,41,44,45,46,47]
GYM-B	#31	/	C_32_H_45_NO_5_	523.3	5.1	Clams (*Ruditapes decussatus*); Mussels (*M. galloprovincialis*)	[25,41,42]
GYM-C	#32	/	C_32_H_45_NO_5_	523.3	/	Clams (*Ruditapes decussatus*)	[25,42]
10-O-dodecanoyl-GYM-A	#89	12:0	C_44_H_67_NO_5_	689.5	/	Clams (*Ruditapes decussatus*)	[42]
10-O-dodecenoyl-GYM-A	#90	12:1	C_44_H_65_NO_5_	687.5	/	Clams (*Ruditapes decussatus*)	[42]
10-O-tetradecanoyl GYM-A	#91	14:0	C_46_H_71_NO_5_	717.5	/	Clams (*Ruditapes decussatus*)	[42]
10-O-tetradecenoyl GYM-A	#92	14:1	C_46_H_69_NO_5_	715.5	/	Clams (*Ruditapes decussatus*)	[42]
10-O-tetradecatrienoyl-GYM-A	#93	14:3	C_46_H_65_NO_5_	711.5	/	Clams (*Ruditapes decussatus*)	[42]
10-O-pentadecanoyl-GYM-A	#94	15:0	C_47_H_73_NO_5_	731.5	/	Clams (*Ruditapes decussatus*); Mussels (*M. galloprovincialis*)	[41,42]
10-O-pentadecenoyl-GYM-A	#95	15:1	C_47_H_71_NO_5_	729.5	/	Clams (*Ruditapes decussatus*); Mussels (*M. galloprovincialis*)	[41,42]
10-O-hexadecanoyl-GYM-A	#96	16:0	C_48_H_75_NO_5_	745.6	/	Clams (*Ruditapes decussatus, Antigona lamellaris*); Pen shell (*Atrina pectinata*); Mussels (*M. galloprovincialis*)	[41,42]
10-O-hexadecenoyl-GYM-A	#97	16:1	C_48_H_73_NO_5_	743.5	/	Clams (*Ruditapes decussatus*); Mussels (*M. galloprovincialis*)	[41,42]
10-O-hexadecadienoyl-GYM-A	#98	16:2	C_48_H_71_NO_5_	741.5	/	Clams (*Ruditapes decussatus*)	[42]
10-O-hexadecatrienoyl-GYM-A	#99	16:3	C_48_H_69_NO_5_	739.5	/	Clams (*Ruditapes decussatus*)	[42]
10-O-hexadecatetraenoyl-GYM-A	#100	16:4	C_48_H_67_NO_5_	737.5	/	Clams (*Ruditapes decussatus*)	[42]
10-O-heptadecanoyl-GYM-A	#101	17:0	C_49_H_77_NO_5_	759.6	/	Clams (*Ruditapes decussatus*); Mussels (*M. galloprovincialis*)	[41,42]
10-O-heptadecenoyl-GYM-A	#102	17:1	C_49_H_75_NO_5_	757.6	/	Clams (*Ruditapes decussatus*); Mussels (*M. galloprovincialis*)	[41,42]
10-O-heptadecadienoyl-GYM-A	#103	17:2	C_49_H_73_NO_5_	755.5	/	Clams (*Ruditapes decussatus*); Mussels (*M. galloprovincialis*)	[41,42]
10-O-octadecanoyl-GYM-A	#104	18:0	C_50_H_79_NO_5_	773.6	/	Clams (*Ruditapes decussatus, Antigona lamellaris*); Pen shell (*Atrina pectinata*); Mussels (*M. galloprovincialis*)	[41,42]
10-O-octadecenoyl-GYM-A	#105	18:1	C_50_H_77_NO_5_	771.6	/	Clams (*Ruditapes decussatus; Antigona lamellaris*); Pen shell (*Atrina pectinata*); Mussels (*M. galloprovincialis*)	[41,42]
10-O-octadecadienoyl-GYM-A	#106	18:2	C_50_H_75_NO_5_	769.6	/	Clams (*Ruditapes decussatus*); Mussels (*M. galloprovincialis*)	[41,42]
10-O-octadecatrienoyl-GYM-A	#107	18:3	C_50_H_73_NO_5_	767.5	/	Clams (*Ruditapes decussatus*); Mussels (*M. galloprovincialis*)	[41,42]
10-O-octadecatetraenoyl-GYM-A	#108	18:4	C_50_H_71_NO_5_	765.5	/	Clams (*Ruditapes decussatus*); Mussels (*M. galloprovincialis*)	[41,42]
10-O-nonadecanoyl-GYM-A	#109	19:0	C_51_H_81_NO_5_	787.6	/	Clams (*Ruditapes decussatus*)	[42]
10-O-nonadecenoyl-GYM-A	#110	19:1	C_51_H_79_NO_5_	785.6	/	Clams (*Ruditapes decussatus*); Mussels (*M. galloprovincialis*)	[41,42]
10-O-nonadecadienoyl-GYM-A	#111	19:2	C_51_H_77_NO_5_	783.6	/	Clams (*Ruditapes decussatus*); Mussels (*M. galloprovincialis*)	[41,42]
10-O-eicosanoyl-GYM-A	#112	20:0	C_52_H_83_NO_5_	801.6	/	Clams (*Ruditapes decussatus*)	[42]
10-O-eicosenoyl-GYM-A	#113	20:1	C_52_H_81_NO_5_	799.6	/	Clams (*Ruditapes decussatus; Antigona lamellaris*); Pen shell (*Atrina pectinata*); Mussels (*M. galloprovincialis*)	[41,42]
10-O-eicosadienoyl-GYM-A	#114	20:2	C_52_H_79_NO_5_	797.6	/	Clams (*Ruditapes decussatus, Antigona lamellaris*); Pen shell (*Atrina pectinata*); Mussels (*M. galloprovincialis*)	[41,42]
10-O-eicosatrienoyl-GYM-A	#115	20:3	C_52_H_77_NO_5_	795.6	/	Clams (*Ruditapes decussatus*); Mussels (*M. galloprovincialis*)	[41,42]
10-O-eicosatetraenoyl-GYM-A	#116	20:4	C_52_H_75_NO_5_	793.6	/	Clams (*Ruditapes decussatus*); Mussels (*M. galloprovincialis*)	[41,42]
10-O-eicosapentaenoyl-GYM-A	#117	20:5	C_52_H_72_NO_5_	790.5	/	Clams (*Ruditapes decussatus*); Mussels (*M. galloprovincialis*)	[41,42]
10-O-heneicosanoyl-GYM-A	#118	21:0	C_53_H_85_NO_5_	815.6	/	Clams (*Ruditapes decussatus*)	[42]
10-O-heneicosenoyl-GYM-A	#119	21:1	C_53_H_83_NO_5_	813.6	/	Clams (*Ruditapes decussatus*)	[42]
10-O-heneicosadienoyl-GYM-A	#120	21:2	C_53_H_81_NO_5_	811.6	/	Clams (*Ruditapes decussatus*)	[42]
10-O-heneicosatrienoyl-GYM-A	#121	21:3	C_53_H_79_NO_5_	809.6	/	Clams (*Ruditapes decussatus*)	[42]
10-O-heneicosatetraenoyl-GYM-A	#122	21:4	C_53_H_77_NO_5_	807.6	/	Clams (*Ruditapes decussatus*)	[42]
10-O-heneicosapentaenoyl-GYM-A	#123	21:5	C_53_H_75_NO_5_	805.6	/	Mussels (*M. galloprovincialis*)	[41]
10-O-docosanoyl-GYM-A	#124	22:0	C_54_H_87_NO_5_	829.7	/	Clams (*Ruditapes decussatus*)	[42]
10-O-docosenoyl-GYM-A	#125	22:1	C_54_H_85_NO_5_	827.6	/	Clams (*Ruditapes decussatus*)	[42]
10-O-docosadienoyl-GYM-A	#126	22:2	C_54_H_83_NO_5_	825.6	/	Clams (*Ruditapes decussatus, Antigona lamellaris*); Pen shell (*Atrina pectinata*); Mussels (*M. galloprovincialis*)	[41,42]
10-O-docosatrienoyl-GYM-A	#127	22:3	C_54_H_81_NO_5_	823.6	/	Clams (*Ruditapes decussatus*); Mussels (*M. galloprovincialis*)	[41,42]
10-O-docosatetraenoyl-GYM-A	#128	22:4	C_54_H_79_NO_5_	821.6	/	Clams (*Ruditapes decussatus*); Mussels (*M. galloprovincialis*)	[41,42]
10-O-docosapentaenoyl-GYM-A	#129	22:5	C_54_H_77_NO_5_	819.6	/	Clams (*Ruditapes decussatus*); Mussels (*M. galloprovincialis*)	[41,42]
10-O-docosahexaenoyl-GYM-A	#130	22:6	C_54_H_75_NO_5_	817.6	/	Clams (*Ruditapes decussatus*); Mussels (*M. galloprovincialis*)	[41,42]
10-O-tetracosanoyl-GYM-A	#131	24:0	C_56_H_91_NO_5_	857.7	/	Clams (*Ruditapes decussatus*)	[42]
10-O-tetracosenoyl-GYM-A	#132	24:1	C_56_H_89_NO_5_	855.7	/	Clams (*Ruditapes decussatus*)	[42]
10-O-tetracosapentaenoyl-GYM-A	#133	24:5	C_56_H_81_NO_5_	847.6	/	Clams (*Ruditapes decussatus*); Mussels (*M. galloprovincialis*)	[41,42]
10-O-tetracosahexaenoyl-GYM-A	#134	24:6	C_56_H_79_NO_5_	845.6	/	Clams (*Ruditapes decussatus*); Mussels (*M. galloprovincialis*)	[41,42]
O-octadecanoyl-GYM-B	#135	18:0	C_50_H_79_NO_6_	789.6	/	Clams (Ruditapes decussatus)	[42]
O-octadecanoyl-GYM-C	#136	18:0	C_50_H_79_NO_6_	789.6	/	Clams (Ruditapes decussatus)	[42]

^1^ LogP predicted from the chemical structure of molecules using the ACD/Labs platform. Octanol/water partition coefficient are predicted using algorithms.
